# Influence of Dosing Regimen and Adjuvant Type on the Immunogenicity of Novel Recombinant Zika Virus-Like Particles

**DOI:** 10.1128/spectrum.02885-22

**Published:** 2022-12-21

**Authors:** Gabriela Brzuska, Boguslaw Szewczyk, Ewelina Krol

**Affiliations:** a Department of Recombinant Vaccines, Intercollegiate Faculty of Biotechnology, University of Gdansk and Medical University of Gdansk, Gdansk, Poland; Regional Centre for Biotechnology

**Keywords:** Zika virus, adjuvants, antigen specificity, vaccines, virus-like particles

## Abstract

Zika virus (ZIKV) is a reemerging mosquito-borne flavivirus that causes febrile illness and is also linked to Guillain-Barré syndrome as well as to microcephaly in newborns. Due to the risk of fetuses developing microcephaly, ZIKV is a serious problem for pregnant women. Although different types of vaccine antigens have been investigated, there is still no approved vaccine that prevents ZIKV. The aim of this study was to produce a potential anti-Zika virus vaccine candidate based on virus-like particles (VLPs) in mammalian cells and to analyze the role of dosing regimen and adjuvant type on the immunogenicity of the obtained antigen. Novel recombinant VLPs (F2A) were designed by introducing the optimized signal sequence of prM protein and by adding a self-cleavage peptide 2A between proteins prM and E. These modifications improved the formation of the glycoprotein E dimer. It has been shown that the increasing dosing regimen generates a significantly higher titer of antibodies; however, the adjuvant type does not affect this process. Sera from mice immunized using an increasing dosing schedule also showed higher neutralization activity against both Zika strains (H/PAN/2016/BEI-259634, a pandemic strain belonging to Asian lineage, and MR766, a reference strain from African lineage). In summary, this is the first report showing the influence of vaccination schedules and adjuvants on the immunogenicity of ZIKV virus-like particles.

**IMPORTANCE** Considering the transmission of ZIKV and the risk of another epidemic as well as the neurological complications that follow ZIKV infection, the virus remains a serious problem for the human population, especially pregnant women. Therefore, there is a great need to develop new effective vaccine candidates. Although different types of vaccine antigens have been used in preclinical studies worldwide, there is still no approved vaccine to prevent ZIKV. VLPs are among the most potent antigens, but to use VLPs, adjuvants must be added to the formulation and appropriate administration must be performed. In this study, we show for the first time the influence of vaccination schedules and adjuvants on the immunogenicity of recombinant ZIKV VLPs. The obtained results can be used in new vaccine designs not only against ZIKV but also against other important viral pathogens.

## INTRODUCTION

Zika virus (ZIKV) is a mosquito-borne human pathogen that was discovered in 1947 in West Africa (1). For many years, ZIKV infections were sporadic, then the first large outbreaks occurred in 2007 in the region of Micronesia and in 2013 in French Polynesia, at which time ZIKV was first connected with the neurological disorder Guillain-Barré syndrome (2, 3). Then, ZIKV quickly spread through the Pacific region to South America in 2015 to 2016, causing an epidemic in those regions. In Brazil, the number of ZIKV cases was estimated to be more than 1,300,000 (World Health Organization, 2016). Importantly, the epidemic was followed by a striking increase in the cases of microcephaly in newborns (4, 5). To date, 89 countries have reported Zika virus transmission caused by the spread of *Aedes* spp. mosquitoes, although ZIKV can also be transmitted via other routes, i.e., blood transfusion, from mother to child, and via sexual contact. The geographical range of mosquitoes is still expanding due to climate changes, and there are new reports of active transmission of flaviviruses, e.g., West Nile virus, Usutu virus, dengue virus, and Zika virus (6). One of the latest reports described new cases of active vector transmission of ZIKV in France (7, 8). Although the overall number of ZIKV infections is now declining; e.g., in 2020 in Brazil, there were over 3,500 confirmed cases versus 215,000 in 2016, ZIKV is still actively circulating in Latin America and Asia (9). Recent studies have shown new ZIKV African strains have emerged in mosquitoes and primates, and these strains are genetically different from pandemic strains by 15%; in addition, these strains are more infectious and pathogenic to fetuses (10–12).

Although many efforts have been made to develop an effective vaccine or antiviral therapy, no vaccine has been approved (13). Considering the transmission of ZIKV and the risk of another epidemic as well as neurological complications following ZIKV infection, ZIKV remains a serious problem for the human population, especially pregnant women. Therefore, there is a need to study ZIKV pathology in detail and to develop new effective vaccine candidates.

Virus-like particles (VLPs) are among the strategies for developing vaccine antigens. Naturally, two envelope glycoproteins of ZIKV, prM and E, are able to assemble into VLPs and are further secreted to the cell culture medium when expressed together in eukaryotic cells (14). However, the level of VLPs secretion may be low, and therefore different strategies, such as exchanging the signal sequence of prM protein or the transmembrane domains of E protein to analogous regions of Japanese encephalitis virus (JEV), have been used (15, 16). Additionally, capsid protein (C) may be added to the VLPs construct to facilitate production; however, this strategy requires coexpression with viral protease to cleave the C protein from prM and E proteins. The immunogenic potential of ZIKV VLPs has been demonstrated in animal models, and it was shown that compared to inactivated virus, these structures are more immunogenic (17, 18). The dosage of VLPs used in these studies ranged from 1 to 25 μg of total protein content. Furthermore, it was shown that the formulation of ZIKV VLPs with various adjuvants (e.g., aluminum hydroxide, squalene-based oil-in-water nanoemulsion) resulted in an increase in neutralizing antibody titers in comparison to nonadjuvanted VLPs when administered in a two-dose regimen via the intramuscular route.

In this study, we analyzed the influence of VLPs dosing and immunization regimens on the immunogenicity of ZIKV VLPs. For this purpose, we designed novel recombinant VLPs by modifying the signal sequence of prM and introducing a self-cleavage peptide to increase the secretion of VLPs. VLPs were produced in a mammalian expression system and further purified using a two-step chromatography process. Next, we examined the influence of the dosing regimen—three increasing or decreasing doses of VLPs in combination with a squalene-based oil-in-water nanoemulsion adjuvant—in a mouse model on the antibody response. The selected dosing regimen of VLPs was also tested with another adjuvant, aluminum hydroxide in combination with monophosphoryl lipid A (MPLA). The results show superiority of the increasing dosing regimen in the generation of an antibody response against Zika virus; however, the adjuvant type did not significantly influence the immunogenicity of VLPs. Moreover, despite the numerous studies on the potency of VLPs as vaccine antigens against ZIKV, this is the first report of the influence of vaccination schedules and adjuvants on their immunogenicity.

## RESULTS

**FIG 1:**
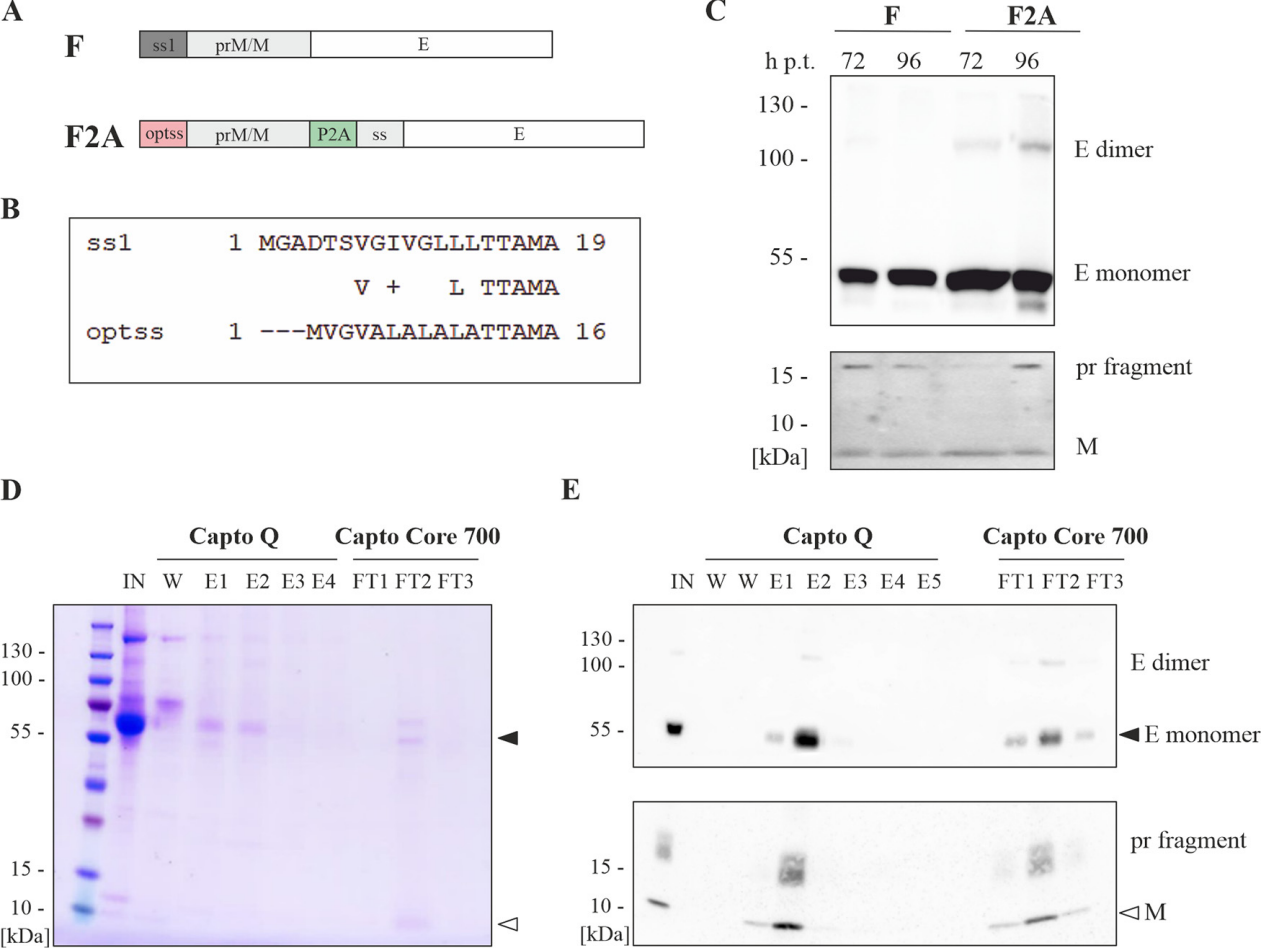
Design, expression and purification of recombinant F2A VLPs. (A) Schematic design of F2A VLP genes in comparison to wild-type VLPs (F); ss1- wild-type signal sequence of prM/M protein, optss- optimized signal sequence of prM/M protein, P2A- the self-cleavage peptide 2A, ss - wild-type signal sequence of E protein. (B) The alignment of ss1 and optss amino acid sequences. (C) Western blotting of E and prM/M protein expression in cell culture medium. 293T cells were transfected with pcDNA 3.1 plasmids encoding genes of F VLPs or F2A VLPs and then 72 and 96 h posttransfection (h p.t.) samples of cell culture medium (20 μg of total protein content of each sample) were collected and used to analyze the protein expression of E and prM/M proteins expression in nonreducing conditions using monoclonal 4G2 antibody and anti-prM polyclonal antibody. (D) Coomassie stained 4 to 20% SDS-PAGE gel of fractions collected during purification of F2A VLPs. (E) Western blotting of E and prM/M proteins in fractions collected during purification steps. IN- input sample of cell culture supernatant, W- wash fractions from the first step purification on the Hitrap Capto Q column, E1-5- elution fractions Hitrap Capto Q column, FT1-3- flow-through fractions from second step purification on the HiScreen Capto Core 700 column.

To increase the production of ZIKV VLPs in mammalian cells, novel recombinant VLPs (F2A) were designed by exchanging the native signal sequence of prM protein with an artificial, optimized signal sequence and introducing a self-cleavage peptide 2A from porcine teschovirus-1 between prM and E protein (Fig. 1A and B). The wild-type VLPs (F) and the F2A VLPs in mammalian cells were then expressed to compare the expression of prM and E proteins, and 293T cells were transfected with plasmid vectors (pcDNA3.1) encoding both types of VLPs. The transfection was performed at 37°C for 72 and 96 h posttransfection. Next, the cell culture supernatants were harvested, and prM/M and E protein expression was evaluated by Western blotting (Fig. 1C). The F2A VLPs showed a higher level of the E protein monomeric form (~55 kDa), and more importantly, E dimers (~110 kDa) were more abundant than in the F VLPs. Both types of VLPs showed similar levels of pr fragment (~17 kDa) and M protein (~9 kDa).

Moreover, the immunogenic properties of both types of VLPs were compared in animal studies. For that purpose, VLPs were purified from cell culture medium using polyethylene glycol 6,000 precipitation, followed by size exclusion chromatography. The purified particles were used in combination with AddaVax adjuvant for the immunization of mice (subcutaneous route with 25, 15, and 10 μg doses and a 2-week interval). The obtained results demonstrated the superiority of the F2A VLPs in the induction of anti-E antibodies in the sera from immunized animals, as well as the stronger neutralizing activity of immune sera (Fig. S1). Therefore, only F2A VLPs were used for further experiments.

It has been proven that a 28°C incubation temperature after transfection can also increase ZIKV protein expression and favor the formation of E dimers (19). However, to further maximize VLPs production, we combined this approach with a sodium butyrate (NaBut) treatment of transfected cells. Sodium butyrate is a histone deacetylase inhibitor known to enhance glycoprotein expression or the production of lentiviruses in mammalian cells (20–22). This modification increased the yield of secreted prM/M and E proteins by ~3-fold (Fig. S2).

In the next step, F2A VLPs were produced using the above protocol and then purified using a simple chromatographic process. The cell culture medium was harvested and clarified via centrifugation and filtration, and then F2A VLPs were subsequently purified using a two-step chromatographic process with anion exchange and multimodal chromatography. The first step was performed using a HiTrap Capto Q column to capture the F2A VLPs, which were then eluted and loaded directly on the second HiScreen Capto Core 700 column. With this column, the F2A VLPs were excluded from the shell of the resin and were recovered in the flow-through fractions while the cell culture proteins remained bound to the resin. The purity of the F2A VLPs and the content of prM/M and E proteins were evaluated in fractions collected during the purification process using Coomassie staining and immunoblotting, respectively (Fig. 1D and E). The F2A VLPs were efficiently purified using this two-step method. Two forms of E protein (monomer and dimer) were observed, and the pr fragment and M protein were also detected. The protein concentration in the final preparation of the F2A VLPs was approximately 0.1 to 0.2 mg/mL according to the Bradford method.

**FIG 2:**
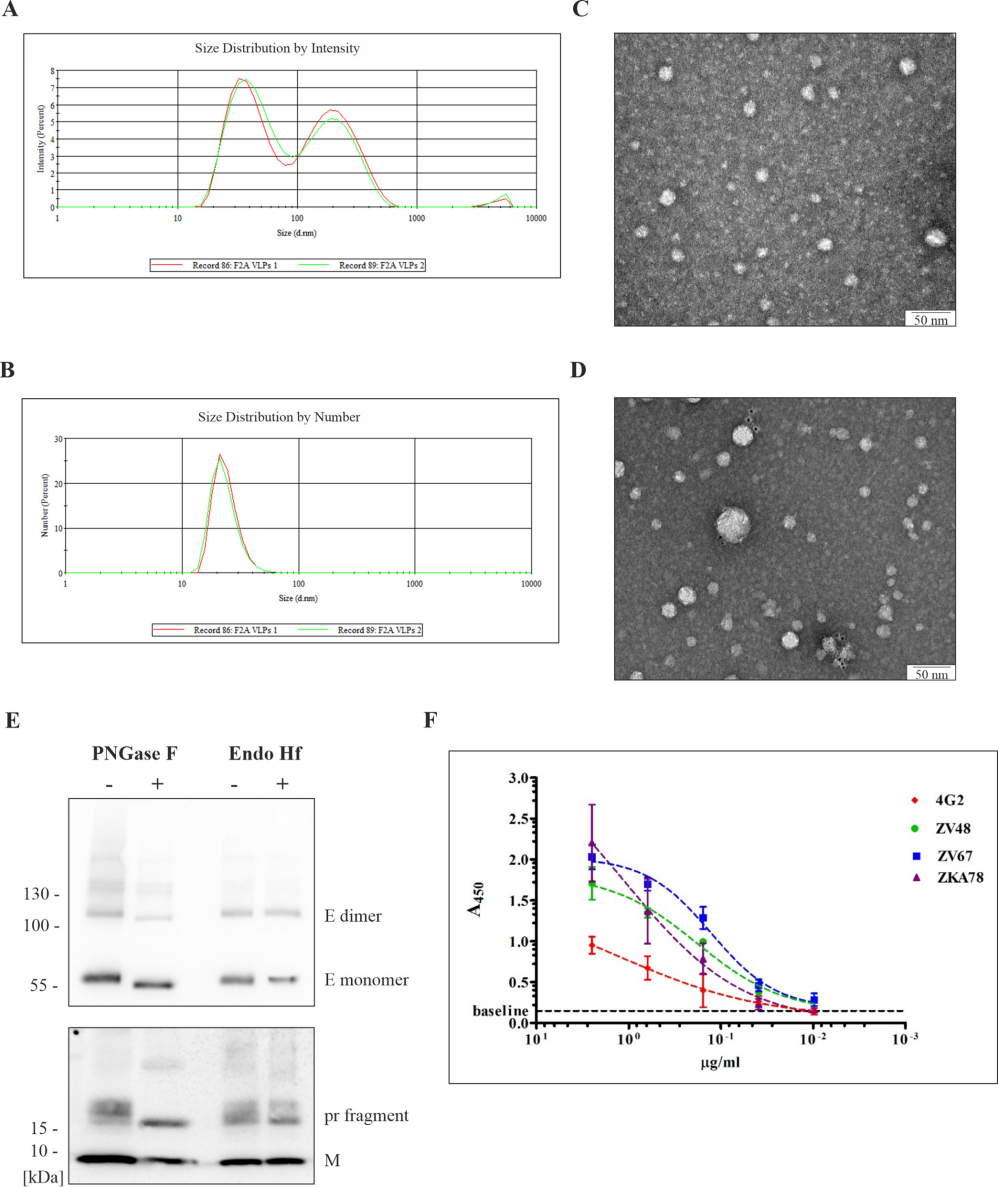
Morphology and antigenicity analysis of F2A VLPs. (A and B) Dynamic light scattering (DLS) analysis of the F2A VLPs particle size distribution by intensity (A) and by number (B). (C and D) TEM analysis of the F2A VLPs following negative staining with 2% uranyl acetate (C) and after immunogold labeling with the ZV67 antibody (D); scale bar 50 nm. (E) Immunoblots of the prM/M and E protein mobility shifts in SDS-PAGE gel after PNGase F and Endo Hf treatment. Treatment with F2A VLPs (2 μg of total protein content) was performed under denaturing conditions. The blot of the E protein was incubated with anti-DIII monoclonal antibody, and the blot of the prM/M protein was probed with anti-prM polyclonal antibody. (F) ELISA of monoclonal antibodies (4G2, ZV48, ZV67 and ZKA78) binding to F2A VLPs. The plate was coated with serially diluted F2A VLPs, and then diluted antibodies were added. Two independent experiments were performed in triplicate, and the mean absorbance at 450 nm is shown in the diagram. Error bars indicate standard deviations.

Next, F2A VLPs were characterized in terms of morphological properties and antigenicity. The presence of F2A VLPs in the final preparation was evaluated using dynamic light scattering (DLS) and transmission electron microscopy (TEM) (Fig. 2). The DLS analysis by intensity showed two populations of particles, with mean hydrodynamic diameters of ~35 nm and ~260 nm; however, the peak of larger particles exhibited a lower intensity (Fig. 2A). The DLS analysis by number showed one peak with a mean hydrodynamic diameter of ~40 nm (Fig. 2B). Transmission electron microscopy analysis confirmed the presence of VLPs (Fig. 2C). Particles with different sizes (~60 nm and ~30 nm) were also observed after negative uranyl staining. Furthermore, the presence of VLPs in the final preparation was confirmed by an immunogold labeling method using the ZV67 antibody, which was directed against the epitope in domain III of the E protein (Fig. 2D).

As genetic modifications were introduced into the prME glycoprotein gene cassette, protein N-glycosylation and the antigenicity of the F2A VLPs were analyzed. Both prM and E are glycoproteins, and each contain one N-glycosylation site in the pr fragment (N69) and in the DI of E (N154), respectively (23–25). N-glycosylation is essential for the ZIKV replication cycle and plays a pivotal role in viral protein folding, protein trafficking, and virion assembly. The N-glycosylation profile of prM/M and E glycoproteins was evaluated using treatment with the following glycosidases: PNGase F (cleaves all N-glycans) and Endo Hf (cleaves immature high-mannose N-glycans) (Fig. 2E). PNGase F treatment with the F2A VLPs resulted in the shift of E protein migration in the SDS-PAGE gel (both monomeric and dimeric forms), as well as in the migration of the pr fragment, confirming N-glycosylation of both proteins. The Endo Hf digestion of F2A VLPs did not affect the mobility of glycoproteins, indicating that N-glycans did not contain high-mannose structures. Moreover, we analyzed the antigenicity of the produced F2A VLPs with the following neutralizing antibodies, which were directed against conformational epitopes in the E protein: the fusion loop in domain II (4G2), the C-C’ loop in DIII (ZV48), the lateral ridge in DIII of mature particles (ZV67), and the epitope in DI/DII (ZKA78) (Fig. 2F). ZV48 and ZV67 exhibit high neutralizing activity and no cross-reactivity with other flaviviruses (26). Although the 4G2 and ZKA78 antibodies show neutralizing potential, they also enhanced the ZIKV infection in *in vitro* studies (27). The binding of the 4G2 antibody to particles was the weakest compared to that of all other examined antibodies. In summary, the F2A VLPs retained the N-glycosylation of prM/M and E proteins, and 4 monoclonal antibodies against E protein interacted with particles.

**FIG 3:**
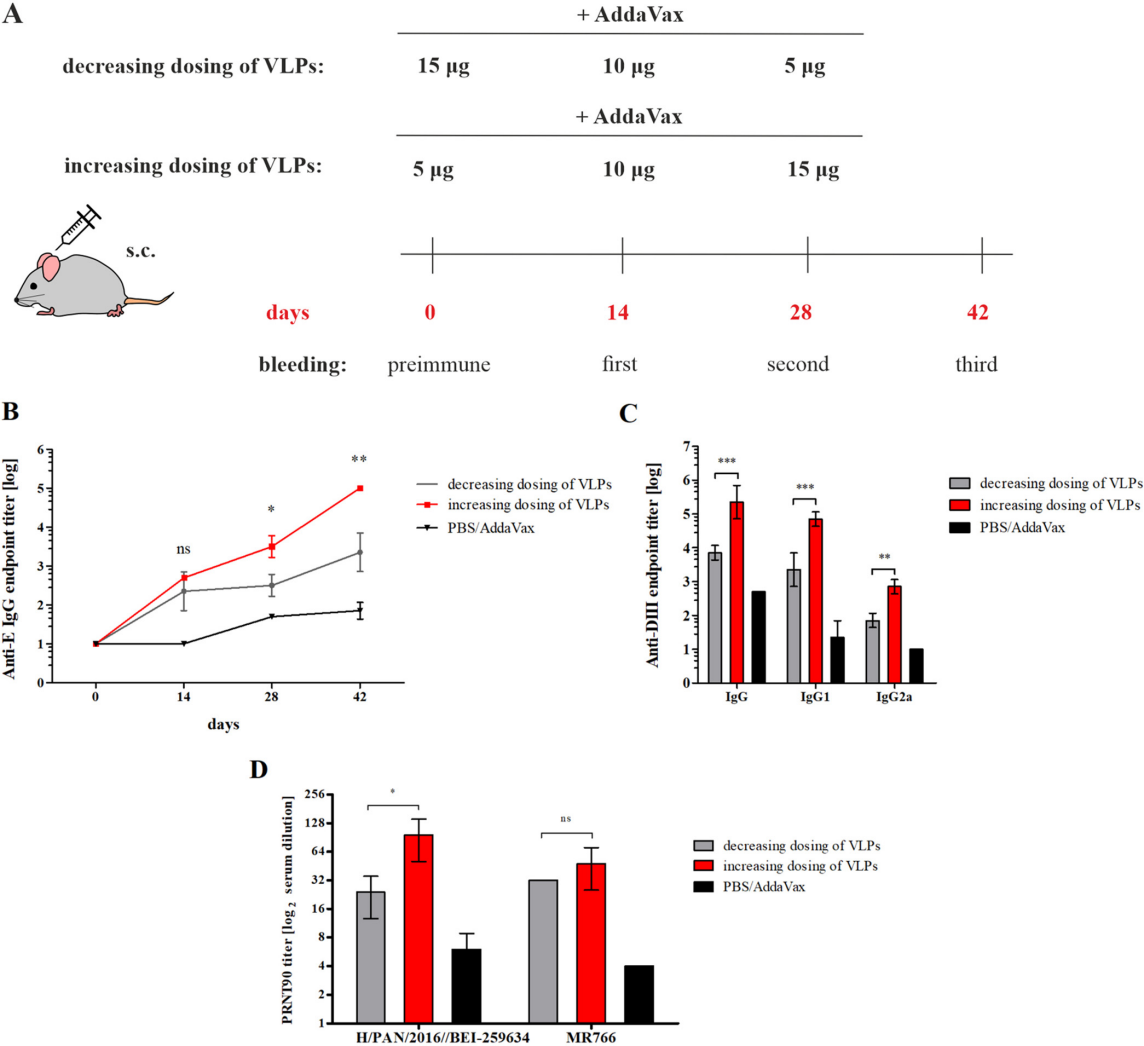
The influence of dosing regimens on the immunogenicity of VLPs. (A) Schematic illustration of vaccination regimens and the timeline of the experiment. (B) The analysis of antibody levels against E protein in sera from immunized mice. The endpoint titers of anti-E IgG in sera collected at different time points were determined using ELISA. The analysis was performed twice, and mean values are presented on the graph; error bars indicate standard deviations. Statistical significance was calculated using two-way ANOVA with Bonferroni posttest. *P* > 0.05; ns, *, *P* < 0.05; *, *P* < 0.01. (C) The analysis of IgG/IgG1/IgG2a antibodies against domain III of ZIKV. The endpoint titers of anti-DIII IgGs, IgG1 and IgG2a in sera collected on day 42 were determined using an ELISA. The analysis was performed twice, and the mean values are presented on the graph; error bars indicate standard deviations. Statistical significance was calculated using two-way ANOVA with Bonferroni posttest. **, *P* < 0.01; ***, *P* < 0.001. (D) Analysis of the serum neutralizing activity against ZIKV. The sera neutralizing activity was measured using a plaque reduction neutralization test (PRNT) against the following Zika virus strains: H/PAN/2016/BEI-259634 and MR766. The diagram shows the PRNT90 values, which are the highest dilutions of sera that resulted in at least 90% reduction in ZIKV plaques. The analysis was performed twice, and the mean values are presented on the graph; error bars indicate standard deviations. Statistical significance was calculated using two-way ANOVA with Bonferroni posttest. *, *P* > 0.05; **, *P* < 0.05.

Next, the potential of F2A VLPs as vaccine antigens was assessed. Moreover, the influence of the dosing regimen on the immunogenicity of F2A VLPs was evaluated. Typically, in a multiple vaccination schedule, 2 to 3 doses of the same antigen are administered or decreasing doses of antigen are used. In contrast, for human immunodeficiency virus antigens, it has been shown that subcutaneous immunization with multiple increasing doses of antigens leads to an increase in neutralizing antibody levels, which results from a longer immunogen retention in the lymph nodes than that of immunization with the same doses (28). Two groups of BALB/c mice (*n* = 6) were immunized via the subcutaneous route (s.c.) three times, 2 weeks apart, with three increasing or decreasing doses of F2A VLPs (5, 10 or 15 μg of total protein content) formulated with AddaVax adjuvant (a squalene-oil-in water nanoemulsion, analog of MF59 adjuvant). This adjuvant is capable of stimulating both cellular and antibody responses (29). A negative-control group (*n* = 3) received PBS buffer plus adjuvant. Serum samples were collected 2 weeks after each immunization and were used to evaluate the antibody response (Fig. 3A). To assess the generation of antibodies over time, the ectodomain of E glycoprotein was used as an antigen in the ELISA (Fig. 3B). Both vaccine formulations resulted in the generation of anti-E antibodies. The level of these antibodies was very low after the first dose for each tested vaccine formulation and increased as expected after each boost dose. The highest increase in the antibody titer was observed in sera from the group of mice immunized with the increasing dosing regimen. Moreover, the anti-E antibody level in the sera collected after the second boost dose was ~100 times higher for the group immunized with increasing doses of VLPs than for the other tested vaccination regimen. Next, the neutralizing activity of collected mouse sera (day 42) against Zika virus was evaluated. The plaque reduction neutralization assay with two strains—a pandemic strain belonging to the Asian lineage, H/PAN/2016/BEI-259634, and a reference strain from the African lineage, MR766—was performed (Fig. 3D). Sera from mice immunized using the increasing dosing schedule showed higher neutralization activity than that of serum from mice immunized with the decreasing dosing schedule against both strains, although the difference between PRNT90 values for MR766 was not significant. The cellular response was also evaluated using IFNγ ELISpot on peptide-stimulated murine splenocytes; however, no IFNγ secretion was observed (data not shown). Therefore, the endpoint titers of the whole IgG antibody fraction and two subclasses, IgG1 (Th2-associated) and IgG2a (Th1-associated), against the DIII of the E protein were assessed (Fig. 3C). Immunization of mice with increasing doses of F2A VLPs resulted in higher generation of the IgG response against the DIII of E glycoprotein, and it increased the levels of IgG1 and IgG2a. However, the level of IgG2a was much lower than that of IgG1, regardless of the tested dosing regimen; thus, it can be concluded that the antibody response to VLPs may show a Th2-polarized profile.

**FIG 4:**
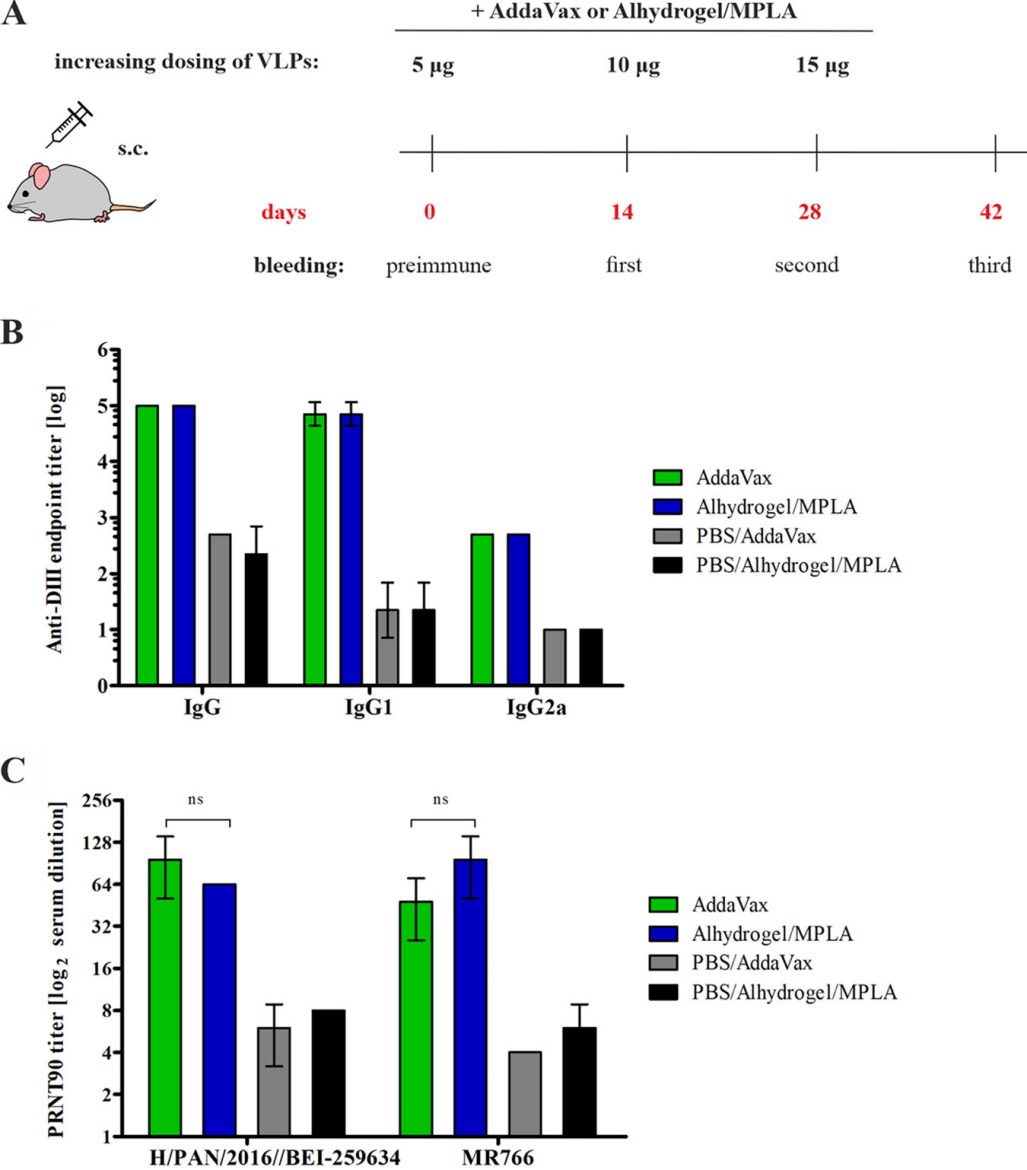
Impact of the adjuvant type on the immunogenicity of VLPs. (A) Schematic illustration of the immunization design and the timeline of the experiment. (B) The analysis of IgG/IgG1/IgG2a antibodies against domain III of ZIKV. The endpoint titers of anti-DIII IgGs, IgG1 and IgG2a in sera collected on day 42 were determined using an ELISA. The analysis was performed twice, and the mean values are presented on the graph; error bars indicate standard deviations. (C) Analysis of the serum neutralizing activity against ZIKV. Sera neutralizing activity was measured using a plaque reduction neutralization test (PRNT) against two Zika virus strains: H/PAN/2016/BEI-259634 and MR766. The diagram shows the PRNT90 values, which are the highest dilutions of sera that resulted in at least 90% reduction in ZIKV plaques. The analysis was performed twice, and the mean values are presented on the graph; error bars indicate standard deviations. Statistical significance was calculated using two-way ANOVA with Bonferroni posttest. *P* > 0.05: ns.

In the final step, the influence of adjuvant type on the immunogenic potential of F2A VLPs was examined. We compared AddaVax with another type of adjuvant system, Alhydrogel/MPLA. This system is based on aluminum hydroxide, which stimulates a strong antibody response (Th2-type response) and synthetic monophosphoryl lipid A (MPLA) toll-like receptor 4 (TLR4) agonist, contributing to the cellular (Th1- type) response (30, 31). The F2A VLPs were formulated with these adjuvants and used for mouse immunization according to the increasing dosing schedule (Fig. 4A), and the cellular and antibody response was examined. Again, no T-cell response was observed in the IFNγ ELISpot assay. The levels of IgG, IgG1, and IgG2a against DIII were the same for groups immunized with each of the adjuvant types (Fig. 4B). The neutralization activity of sera against ZIKV was comparable for both adjuvants (Fig. 4C).

## DISCUSSION

Due to climate change, vector-borne flaviviruses, including Zika virus, are constantly spreading, and new viral strains are emerging. Given the risk of neurological complications in fetuses and newborns after ZIKV infection, the development of a safe and effective vaccine remains a high priority.

The main objective of the study was to evaluate the impact of the VLPs dosing regimen and adjuvant type on the antibody response. For this purpose, we designed novel recombinant ZIKV VLPs (F2A VLPs) to facilitate production in eukaryotic cells. We introduced the following changes in the sequence of the prM and E protein cassette: the optimized signal sequence of the prM protein and the inclusion of a self-cleavage peptide (P2A) before a signal sequence of the E protein. Other groups reported the use of various signal sequences, including the JEV prM protein or the IL-2 signal sequence with ZIKV VLPs, to increase VLPs secretion into the culture medium (15, 32). The JEV prM protein signal peptide was also previously used with the VLPs of dengue virus (33). Moreover, substituting one amino acid in the prM signal sequence of ZIKV led to enhanced secretion of E protein, which was even higher than the protein level obtained with the use of the JEV signal peptide (16). In the case of the P2A peptide, this is the first report that used P2A in the flavivirus VLPs construct. The peptide was introduced to maximize the separation of prM and E proteins during translation, in addition to the signal peptidases. Previously, a self-cleavage peptide (from food and mouth disease virus, FMDV) was only used to separate C and prM proteins of tick-borne encephalitis virus (34). The P2A peptide used in this study is a derivative of porcine teschovirus-1 and was selected due to the high efficiency of cleavage in the HEK293 cell line (35). Although we did not observe a significant increase in prM/M secretion, the combination of both modifications resulted in increased levels of E protein in both monomeric and dimeric forms. The presence of E protein dimers in VLPs is crucial, as it was shown that compared to wild-type VLPs, VLPs with covalent E dimers were able to elicit antibodies with a higher potential to neutralize ZIKV (36). Next, we produced the F2A VLPs using a transient expression system in 293T cells. The following changes were made in the production protocol: the use of 28°C combined with sodium butyrate supplementation after cell transfection. It was previously shown that lower temperature stabilizes the formation of ZIKV E dimers (19, 37). Moreover, it has recently been reported that sodium butyrate supplementation can be used to increase JEV VLPs expression in the BHK-21 stable cell line (38). The introduced modifications resulted in approximately 3 times higher secretion of E and M proteins in our studies.

Furthermore, we purified the F2A VLPs using a two-step chromatographic process. The most common methods of flavivirus VLPs purification are polyethylene glycol precipitation, diafiltration, and density gradient ultracentrifugation. However, these methods are laborious and difficult to scale up; thus, other methods need to be explored. Our approach involved a combination of anion-exchange chromatography with multimodal chromatography. In the first step, F2A VLPs were captured from the clarified cell culture medium onto HiTrap Capto Q resin; a high concentration of particles occurred, and most host cell proteins were removed.

In the next step, F2A VLPs were further purified with the use of HiScreen CaptoCore 700, in which particles were recovered from the flow-through. A similar strategy was also applied for the purification of YFV wild-type VLPs and other ZIKV VLPs produced in stable HEK293SF-3F6 cell lines; however, the immunogenicity of these particles was not assessed (39). Moreover, recombinant chimeric ZIKV VLPs (prM and E transmembrane domain sequences derived from the MR766 strain, E ectodomain sequence from the SPH2015 Brazilian strain, preceded by the IL-2 signal sequence) were purified using a two-step chromatographic process; however, the first step (multimodal chromatography) and the second step (salt-tolerant interaction chromatography [STIC-PA]), were performed in reverse order and both operated in negative mode (18). Our analysis together with these reports confirm the applicability of these two chromatographic processes for the purification of VLPs with high recovery and high removal of host cell proteins. Two populations of particles with diameters of ~35 nm and ~55 nm were evident by TEM and DLS analysis. VLPs with similar sizes were observed not only for ZIKV but also for tick-borne encephalitis virus, in which VLPs with smaller sizes were identified as mature particles (18, 40).

In the next step, we characterized the antigenic determinants of F2A VLPs. Due to genetic alterations in the sequence of prM and E glycoproteins in the F2A VLPs, the N-glycosylation status was evaluated first. Treatment with PNGase F demonstrated the presence of mature N-glycans on the E protein and the pr fragment. The N-glycosylation of the ZIKV E protein plays a crucial role in the replication cycle, especially in the assembly and secretion of virions, as shown using VLPs as a model (25). Additionally, neutralizing antibodies can recognize both glycosylated and nonglycosylated epitopes on the E protein; therefore, the glycosylation status of VLPs is highly important (41).

Furthermore, the presence of certain epitopes on F2A VLPs was determined. The ELISA analysis demonstrated high binding of F2A VLPs to two neutralizing antibodies (ZV48 and ZV67), which recognize epitopes in the DIII of the E protein, and one antibody (ZKA78) which is directed against an epitope in the DI/DII. A weaker interaction was observed with cross-reactive 4G2 antibody, which recognizes the fusion loop epitope in the E protein. The ZV67 antibody should bind to E protein, regardless of the oligomeric status of E protein in contrast, to other antibodies (26). The epitope for the ZV48 antibody should be hidden in the dimeric form of the E protein; however, the F2A VLPs were well recognized, which may suggest a different conformation of the E protein in the F2A VLPs. These results can also be explained by the existence of two populations of particles. The main objective of the study was to evaluate the dosing regimen and adjuvant type on the immunogenicity of recombinant ZIKV VLPs. We examined the following vaccination schedules: three decreasing or increasing doses of VLPs formulated with oil-in water nanoemulsion adjuvant (AddaVax), which were both administered subcutaneously. The increasing dosing regimen resulted in higher generation of IgG antibodies against E protein during vaccination with over 2-fold higher levels. The enhancement in immune response magnitude after subcutaneous immunization with exponentially increasing doses of antigen has also been reported for human immunodeficiency virus recombinant proteins (28, 42). The increase in the immune response was attributed to sustained antigen availability, as exponentially decreasing doses of antigens resulted in a lower immune response. Although only three increasing doses of VLPs were administered in our study, the antibody response was magnified. In these studies, the increasing dosing regimen also enhanced the levels of IgG1 and IgG2a antibodies against the DIII antigen, although the overall level of IgG2a was low. This suggests that the immune response against VLPs formulations may be Th2-polarized, especially when the cellular response was not detected. In contrast, baculovirus-expressed ZIKV VLPs have been shown to induce a cellular response and Th1-polarized response, which may be due to the difference in the administration route (intramuscular route) (43). Moreover, our studies indicated that the increasing dosing regimen immunization evoked a higher level of neutralizing antibodies against two strains of ZIKV.

The other aspect of this study was to evaluate the impact of adjuvant type on the immune response profile. The following types of adjuvants were compared: MF59-like oil-in water nanoemulsion adjuvant (AddaVax) and aluminum hydroxide/MPLA (Alhydrogel/MPLA). Both adjuvants were used in combination with ZIKV VLPs with different regimens (AddaVax, two doses intramuscularly; and Alhydrogel/MPLA, three doses subcutaneously) and were shown to induce a potent antibody response (17, 36). It is believed that these adjuvants function as antigen depots; however, the clearance of antigen and adjuvant may occur independently, which can decrease the span of immune response (29). Therefore, we examined the effects of both adjuvants on the immunogenicity of the obtained VLPs using the increasing dosing schedule. Both adjuvants failed to induce an IFNγ response to VLPs and the anti-DIII antibody levels were comparable. Moreover, in both cases, the immune response was Th2-polarized. Compared to MF59, the Alhydrogel/MPLA adjuvant in a combination with a recombinant subunit DIII was shown to enhance and generate a more balanced immune response when administered intramuscularly (two doses) (44). In this context it would be noteworthy to compare the immunogenicity of ZIKV or other flavivirus VLPs administered via different routes.

To conclude, a novel strategy for the design of ZIKV VLPs and the production protocol in the mammalian expression system was implemented. The recombinant VLPs could be easily purified via chromatographic methods. Antigenic determinants, such as N-glycosylation, and the display of certain epitopes on the surface of particles, were analyzed. The most important observation that follows from our studies is the crucial role of the dosing regimen, but not the adjuvant type, for the immunogenicity of ZIKV VLPs in a mouse model. In our next study we aim to evaluate the protective efficacy of F2A VLPs in the preclinical infection models.

## MATERIALS AND METHODS

### Gene and plasmid constructs.

The sequences of structural proteins were derived from the ZIKV BeH818995 strain (GenBank: AMA12084.1). The wild-type VLPs construct (F) consists of prM and E proteins preceded by the wild-type signal sequence of prM- transmembrane domains of the capsid protein C region 105 to 122 in the amino acid sequence of the Zika virus polyprotein (ss1). The recombinant F2A VLPs construct was designed by introducing the artificial signal sequence (optss) instead of the prM signal sequence and the P2A self-cleavage peptide between prM and E proteins. The signal sequence optss was an optimized artificial 16 amino acid sequence, which was developed based on the ss1 signal sequence. Sequence optimization was carried out by increasing the number of hydrophobic amino acids, such as alanine, valine, glycine and leucine in the ss sequence and shortening the sequence. The amino-acid similarity of both signal sequences was less than 40%. The sequence encoding the P2A peptide was derived from porcine teschovirus-1 (GenBank: NP_653143.1; 979-997). Additionally, the E protein was preceded by a signal sequence derived from the transmembrane domains of the prM protein. The nucleotide sequences of both constructs were chemically synthesized by GeneArt (Thermo Fisher Scientific).

The synthesized DNA fragments were subcloned into the plasmid vector pcDNA3.1 (Thermo Fisher Scientific) utilizing the BamHI/EcoRI restriction enzyme sites. Plasmids were amplified in TOP10 competent E. coli cells and purified using the Plasmid Midi AX (A&A Biotechnology).

### Cells and viruses.

293T cell line (CRL-3216) and Vero E6 cell line (African green monkey kidney; ATCC CRL-1586) were cultured in Dulbecco’s Modified Eagle’s Medium (d-MEM) (Corning, New York, USA). The A549 cell line (adenocarcinomic human alveolar basal epithelial cells, ATCC CCL-185) was cultured in Minimum Essential Medium Eagle (EMEM) with Earle's Balanced Salt Solution (EBSS) (Lonza). All cells were cultured at 37°C under 5% CO_2_. Both media were supplemented with 2 mM l-glutamine, 8% (vol/vol) heat-inactivated fetal bovine serum (FBS), and 1× Penicillin-Streptomycin (10,000 U/mL) (Gibco).

The following reagent was obtained through BEI Resources, NIAID, NIH: Zika Virus, H/PAN/2016/BEI-259634, NR-50210. The following reagent was obtained through BEI Resources, NIAID, NIH, as a part of the WRCEVA program: Zika Virus, MR 766, NR-50065. Both strains of Zika virus were propagated in Vero E6 cells for 3 to 4 days following a low multiplicity of infection (MOI = 0.01).

### VLPs production.

293T cells were transfected with pcDNA3.1 plasmid vectors encoding F or F2A VLPs constructs using Transporter 5 transfection reagent (Polysciences Europe GmbH) according to the manufacturer’s protocol. Transfection was performed at 37°C for 72 h and 96 h posttransfection. Next, samples were collected from the cell culture medium, and VLPs were precipitated using a solution of 10% polyethylene glycol 6000 (PEG6000) supplemented with 300 NaCl overnight at 4°C. Proteins were pelleted and dissolved in 50 mM Tris/100 mM NaCl/50 mM EDTA buffer. The protein solutions were further used to analyze prM/M and E protein expression.

F2A VLPs for further experiments were produced using an optimized protocol. 16 h posttransfection, the cells were supplemented with 2 mM sodium butyrate, 1× MEM Non-Essential Amino Acids Solution (Gibco), and 0.075% of Sodium Bicarbonate 7.5% solution (Gibco) and were further cultured at 28°C or 37°C. Medium from transfected cells was harvested 96 h posttransfection and clarified via centrifugation at 3,500 × *g* for 10 min at 4°C followed by filtration through a 0.45 μm PVDF filter. The supernatant containing VLPs was stored at 4°C until further analysis or purification.

### SDS-PAGE and Western blot.

The E and prM/M protein contents were analyzed using SDS-PAGE. For all experiments except purification, the same amount of protein in the analyzed samples was used (20 to 40 μg/per well). The total protein content in VLPs samples was quantified using a Quick Start Bradford Protein Assay (Bio-Rad). The samples were incubated at room temperature (RT) in reducing (addition of β-mercaptoethanol) or nonreducing conditions and then loaded on Novex WedgeW 4% to 20% Tris-Glycine polyacrylamide gels (Thermo Fisher Scientific) in Tris-Glycine-SDS running buffer. After electrophoretic separation, the gels were used for Western blotting or Coomassie staining. For Western blotting, the proteins were transferred from gels onto the ROTI PVDF 0.45 μm membrane (Carl Roth) using wet overnight electrotransfer. The membrane was blocked with 5% nonfat milk in TBS-T (TBS buffer with 0.1% Tween 20 [vol/vol]), and ZIKV proteins were detected with the following specific antibodies: mouse monoclonal anti-Flavivirus group antigen antibody (4G2) (Absolute Antibody) (1:2,000 dilution) (used with nonreducing conditions), rabbit monoclonal anti-ZIKV (strain SPH2015) envelope protein (Domain III) antibody (Sino Biological Inc.) (1:5,000 dilution) or rabbit polyclonal Zika virus prM protein antibody (GeneTex International) (1:1,000 dilution), followed by anti-mouse or anti-rabbit HRP-conjugated secondary antibodies (Santa Cruz Biotechnology) (diluted 1:3,000). Next, the blots were developed using Super Signal West Pico Plus Substrate (Thermo Fisher Scientific) and visualized with Chemidoc system Alliance Q9-Series (UVITEC). Coomassie staining of the gel after purification was performed using Imperial Protein Stain (Thermo Fisher Scientific).

### Measurement of E protein levels in the cell culture medium by ELISA.

ELISA plates were coated with clarified cell culture supernatant containing VLPs. After blocking (3% BSA in phosphate-saline buffer [PBS] 0.05% Tween 20 buffer) and washing, rabbit monoclonal anti-ZIKV (strain SPH2015) envelope protein (Domain III) antibody (1:2,000 dilution) was added for 2 h at RT. Next, after washing an anti-rabbit HRP-conjugated secondary antibody (Santa Cruz Biotechnology) (1:1,500) was added. TMB Substrate Solution (Thermo Fisher Scientific) was used for reaction visualization. The reaction was stopped with 0.5 M H_2_SO_4_, and the absorbance was measured at 450 nm with a microplate reader (TECAN).

### VLPs purification.

The F2A VLPs purification process was based on the procedure designed for the purification of yellow fever virus and VLPs with some modifications (39, 45). First, supernatant with F2A VLPs was concentrated using Vivaspin 20, 300,000 MWCO PES. The retentate was further diluted with PBS/50 mM NaCl.

The purification was performed in two chromatographic steps using the anion-exchange chromatography column HiTrap Capto Q, 5 mL (Cytiva) operating in bind-and-elute mode, followed by a multimodal chromatography step using a 4.7 mL HiScreen CaptoCore 700 column (Cytiva) operating in flow-through mode. Both chromatographic steps were performed using a peristaltic pump with a flow rate of 2 mL/min.

In the first step, the HiTrap Capto Q column was equilibrated with PBS/50 mM NaCl, and then the diluted retentate with F2A VLPs was loaded to bind the particles to the resin. Next, the column was washed with PBS/50 mM NaCl and PBS/100 mM NaCl, and F2A VLPs were eluted using PBS/350 mM NaCl. Finally, the column was regenerated by washing with PBS/1 M NaCl, followed by washing with 1 M NaOH.

The second purification step was performed with a multimodal resin. The HiScreen CaptoCore 700 column was equilibrated with PBS/350 mM NaCl, and the eluate from the first step was loaded. The F2A VLPs were recovered in the flow-through fraction. The column was then regenerated by a wash with 30% isopropanol in 1 M NaOH.

The total protein content in the final F2A VLPs preparation was determined using the Bradford method. The purified F2A VLPs were further evaluated by SDS-PAGE, Western blotting, transmission electron microscopy and dynamic light scattering.

### Transmission electron microscopy and immunogold labeling.

The F2A VLPs samples were loaded on carbon-coated 200 mesh nickel grids (Electron Microscopy Science). Negative staining was performed with 2% uranyl acetate. Immunogold labeling was performed according to the protocol of Aurion with some modifications. Briefly, the protocol was performed as follows: particle-loaded grids were blocked with Blocking Solution for Goat Gold Conjugates (Aurion). After washing, the grids were incubated with mouse anti-E-protein DIII (LR) (ZV-67) antibody for 1 h at RT. The labeling was performed with goat anti-mouse IgG conjugated with 6 nm gold particles (Aurion) for 1 h at RT, and then the grids were washed again and fixed with 4% paraformaldehyde. Finally, the grids were stained with 2% uranyl acetate. The samples were analyzed using a transmission electron microscope Tecnai G2 Spirit BioTWIN (FEI) (Faculty of Biology, University of Gdansk, Gdansk, Poland).

### DLS analysis.

To examine the size and distribution of F2A VLPs, the particles were analyzed by Dynamic Light Scattering using a Malvern Zetasizer Nano Z. F2A VLPs samples were diluted in ultrapure water to a protein concentration of 50 μg/mL and analyzed. The hydrodynamic diameter was measurements were performed in duplicates.

### Analysis of N-glycosylation.

The presence of N-glycans on the prM/M and E glycoproteins was analyzed using PNGase F (Thermo Fisher Scientific) and Endo Hf (New England Biolabs) treatment. Two samples of F2A VLPs (2 μg of protein content) were incubated in denaturing buffer, and one sample was treated with PNGase F or Endo Hf, while a second one was an undigested control. The incubation was continued for 16 h at 37°C. After treatment, the samples were analyzed by a mobility shift assay by SDS-PAGE and immunoblotted with anti-domain III or anti-prM antibody as described above.

### Analysis of F2A VLPs antigenicity -binding by specific antibodies.

ELISA plates were coated with purified serially diluted F2A VLPs in PBS buffer pH 7.4. After blocking (3% BSA in PBS- 0.05% Tween 20 buffer) and washing, primary antibodies (2 μg/mL) were added for 2 h at RT. The following different primary antibodies were used: mouse monoclonal anti-flavivirus group antigen antibody (4G2) (Absolute Antibody), mouse monoclonal anti-E-protein DIII antibody (C-C’) (ZV48) (Absolute Antibody), mouse monoclonal anti-E-protein DIII antibody (LR) (ZV67) (Absolute Antibody), and mouse monoclonal anti-Envelope protein antibody (ZKA78) (Absolute Antibody). Next, after washing, an anti-mouse HRP-conjugated secondary antibody (Santa Cruz Biotechnology) (1:1,500) was added. TMB Substrate Solution (Thermo Fisher Scientific) was used for reaction visualization. The reaction was stopped with 0.5 M H_2_SO_4_, and the absorbance was measured at 450 nm with a microplate reader (TECAN).

### Mouse studies.

Two groups of 6 female BALB/c mice (6 to 8 weeks of age) were immunized subcutaneously with a mixture of F2A VLPs and adjuvant AddaVax (InvivoGen) (1:1 vol/vol ratio, final volume 100 μL). Then mice were immunized 3 times in 2-week intervals using the following dosing regimens: decreasing dosing to 15, 10, 5 μg of VLPs or increasing dosing to 5, 10, 15 μg. The total protein content in the VLP antigen for immunization was quantified using a Quick Start Bradford Protein Assay. The mice used as a negative control were immunized with adjuvant and PBS buffer only. Sera were collected on Days 0, 14, 28. On the day 42, the mice were sacrificed, and complete sera were collected for immunological response analysis. For the second study, the following different adjuvants were used: AddaVax and a combination of Alhydrogel 2% (InvivoGen)/Monophosphoryl Lipid A (MPLA) (InvivoGen) using an increasing dosing schedule. The Alhydrogel/MPLA adjuvant was used in the quantity of 200 μg/5 μg. As negative controls, two groups of mice were immunized with PBS and each of the adjuvants. All experiments on animals were conducted by an accredited company (Tri-City Academic Laboratory Animal Centre, Medical University of Gdansk, Gdansk, Poland) in accordance with the current guidelines for animal experimentation. The protocols were approved by the Local Committee on the Ethics of Animal Experiments of the University of Science and Technology in Bydgoszcz (Permit Number: 35/2016 and 17/2020). All surgeries were performed under isoflurane anesthesia, and all efforts were made to minimize suffering.

### Evaluation of serum antibody levels by ELISA.

ELISA plates were coated with recombinant E protein (MyBioSource) or DIII (Sino Biological) antigen (2 μg/mL) in PBS buffer pH 7.4. After blocking (3% BSA in PBS- 0.05% Tween 20 buffer) and washing, serially diluted mouse sera were added for 2 h at RT. Next, after washing, anti-mouse IgG, IgG1 and IgG2a HRP-conjugated secondary antibodies (Santa Cruz Biotechnology, Thermo Fisher Scientific) were added (0.5 μg/mL). TMB Substrate Solution (Thermo Fisher Scientific) was used for reaction visualization. The reaction was stopped with 0.5 M H_2_SO_4_, and the absorbance was measured at 450 nm with a microplate reader (TECAN).

### Plaque reduction neutralization test (PRNT).

To measure ZIKV neutralization by the obtained sera, PRNTs were performed. Briefly, 12-well cell culture plates were seeded with A549 cells to near confluence on the day of ZIKV infection. The sera were heat-inactivated at 56°C for 30 min. Sera were diluted 2-fold in fresh medium and mixed with equal volumes of ZIKV strain H/PAN/2016/BEI-259634 or MR766 containing approximately 50 PFU/well. The mixtures were incubated for 1 h at 37°C and used to infect cells. After 1 h, the inoculum was aspirated, and the cells were overlaid with 1% methylcellulose in MEM medium. The plates were incubated for 4 days at 37°C and stained with 0.5% crystal violet solution in 20% ethanol. Plaques were counted, and PRNT90 was calculated as the highest dilution that resulted in at least a 90% reduction in ZIKV plaques.

### Statistics.

All statistical analyses were performed using GraphPad Prism 5 software. Statistical significance was determined using a two-way ANOVA with Bonferroni posttest.

### Data availability.

The data generated and/or analyzed during this study are available from the corresponding author upon reasonable request.
