# Supplementary material for: Influence of Dosing Regimen and Adjuvant Type on the Immunogenicity of Novel Recombinant Zika Virus-Like Particles
Source: Microbiol Spectr. 2022 Dec 21;11(1):e02885-22. doi: 10.1128/spectrum.02885-22 (PMC9927573; doi:10.1128/spectrum.02885-22)
Supplement: Supplemental file 1 — Supplemental material. Download spectrum.02885-22-s0001.pdf, PDF file, 0.3 MB [file spectrum.02885-22-s0001.pdf]

Influence of dosing regimen and adjuvant type on the immunogenicity of novel recombinant Zika virus-like particles

Running title: Effect of dosing, adjuvant on Zika VLPs immunogenicity

Gabriela Brzuska[a], Boguslaw Szewczyk[a], Ewelina Krol[a#]

[a] Department of Recombinant Vaccines, Intercollegiate Faculty of Biotechnology, University of Gdansk and Medical University of Gdansk, Gdansk, Poland

#Correspondence: Ewelina Krol, Abrahama 58, 80-307 Gdansk;
ewelina.krol@biotech.ug.edu.pl; Tel.: +48-58-523-63-83

Supplementary Data

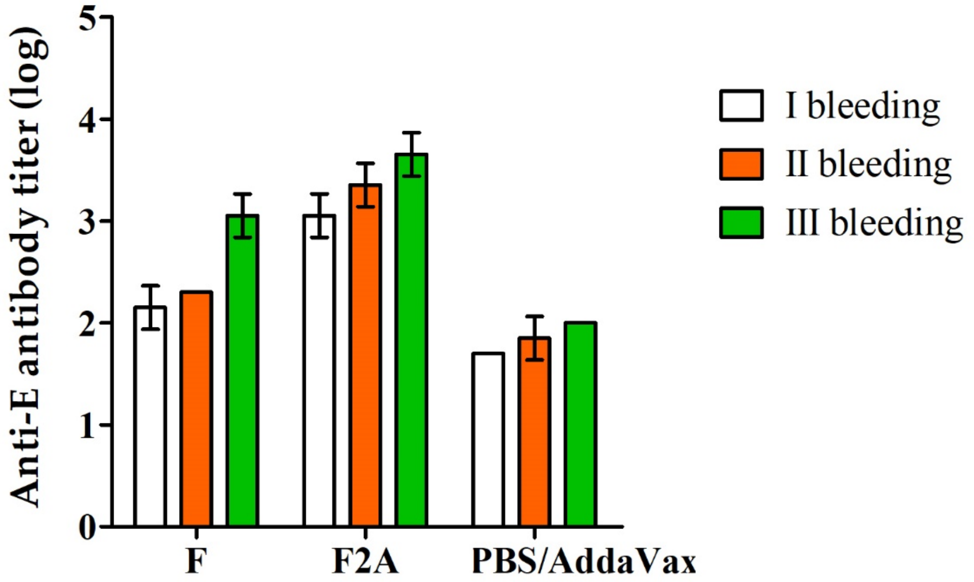

Group	PRNT50 titer (third bleeding)
**F**	**~20**
**F2A**	**~40**
**PBS/AddaVax**	**10**

Figure S1. Comparison of immunogenicity of F and F2A VLPs. Top panel - The analysis of antibody levels against E protein in sera from immunized mice. The endpoint titers of anti-E IgG in sera collected at different time points were determined using ELISA assay. The analysis was performed twice, and mean values are presented on the graph; error bars indicate standard deviations. Bottom panel - Analysis of serum neutralizing activity against ZIKV. Sera neutralizing activity was measured using a plaque reduction neutralization test (PRNT) against Zika virus (strain H/PAN/2016/BEI-259634). The diagram shows the PRNT50 values, which are the highest dilutions of sera that resulted in at least a 50% reduction in ZIKV plaques.

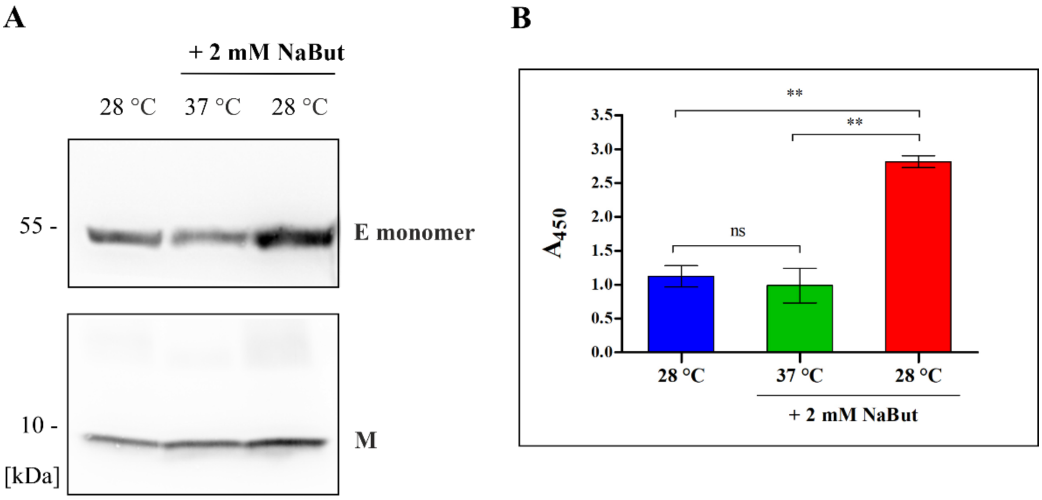

Figure S2. The effect of temperature and sodium butyrate supplementation on F2A VLPs production. Sixteen hours posttransfection, cell cultures were supplemented with 2 mM

sodium butyrate and were further cultured for up to 96 hours at 37 °C or 28 °C. A – Western blot analysis of the E and prM/M protein contents in the clarified cell culture media using anti-DIII and anti-prM/M antibodies, respectively. B – ELISA of the E protein level in the cell culture media. The 96-well plate was coated with the clarified cell culture medium and the E protein was detected using an anti-DIII antibody. Two independent experiments were performed in triplicate, and the mean absorbance at 450 nm is shown in the diagram. Error bars indicate standard deviations. Statistical significance was calculated using one-way ANOVA with Tukey's multiple comparison. $p > 0.05$: ns; $p < 0.01$: **.

Methods

Production and purification of F and F2A VLPs for initial animal studies

F and F2A were produced using a transient expression system in 293T cells. Cells were transfected with plasmids encoding F and F2A VLPs constructs using Transporter™ 5 Transfection Reagent (Polysciences Europe GmbH) according to the manufacturer's protocol. Sixteen hours post transfection, the cells were transferred to 28 °C and further cultured for 72 h. Next, the cell culture media were collected, and clarified using centrifugation (3 500 x g, 10 min), and VLPs were precipitated using a solution of 10% polyethylene glycol 6 000 (PEG6000) supplemented with 300 NaCl overnight at 4 °C. Proteins were pelleted and dissolved in 50 mM Tris/100 mM NaCl/50 mM EDTA buffer overnight. The solutions were clarified by centrifugation (3 500 x g, 10 min) and loaded onto a size exclusion chromatography resin (CL-4B). The elution was performed using PBS buffer with 0.32% trisodium citrate. Fractions with the highest E protein content were pooled and concentrated using Amicon® Ultra 100K filters. The total protein concentration was measured with the Quick Start™ Bradford Protein Assay (Bio-Rad).

Animal studies

Two groups of 6 female BALB/c mice (6-8 weeks of age) were immunized subcutaneously with a mixture of VLPs and the adjuvant - AddaVax (InvivoGen) (1:1 v/v ratio, final volume 100 µl). Mice were immunized 3 times in two-week intervals using the following dosing regimens: decreasing doses of 25, 15 and 10 µg of VLPs. The mice used as a negative control were immunized with adjuvant and PBS buffer only. Sera were collected on Days 0, 14 and 28. On Day 42, the mice were sacrificed, and complete sera were collected for immunological response analysis. As negative controls, two groups of mice were immunized with PBS and the adjuvant.
